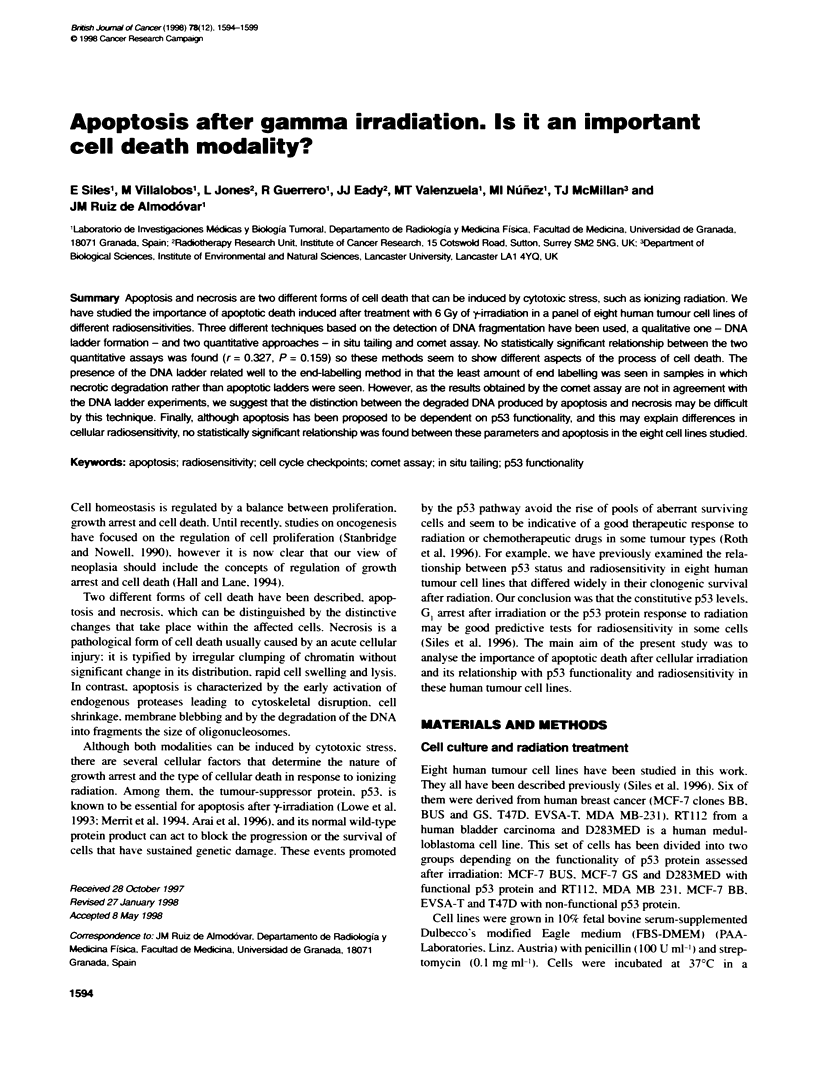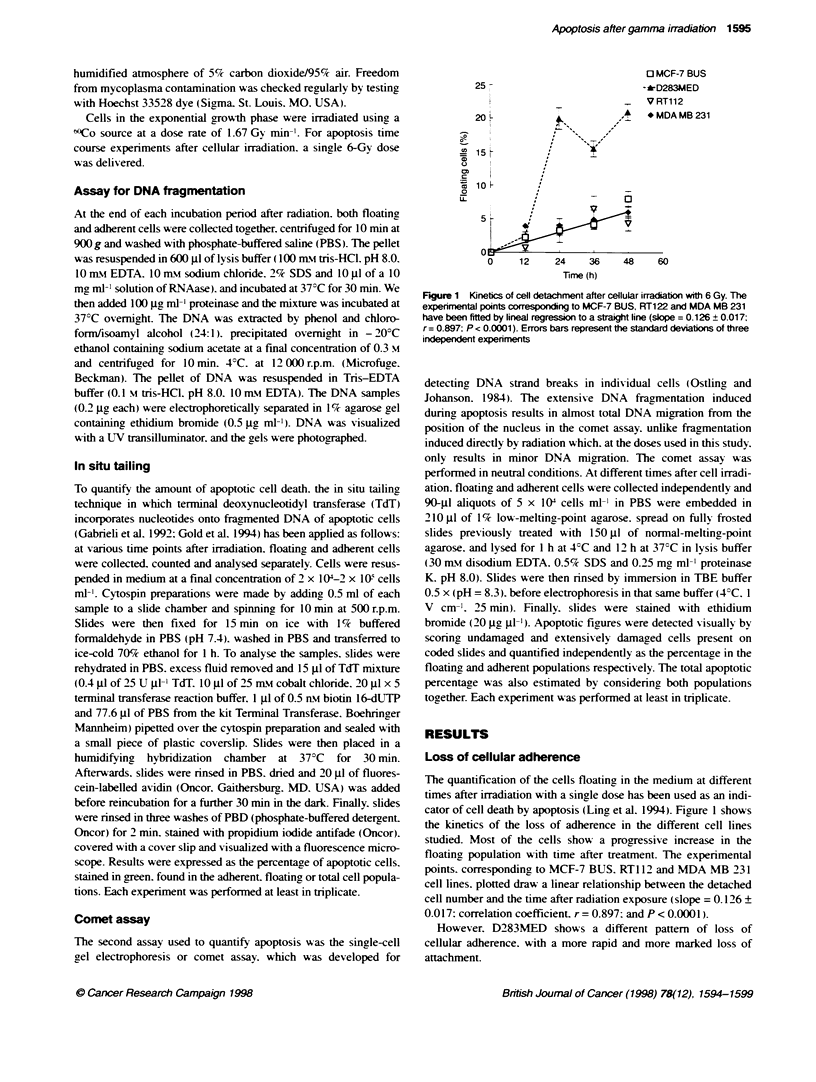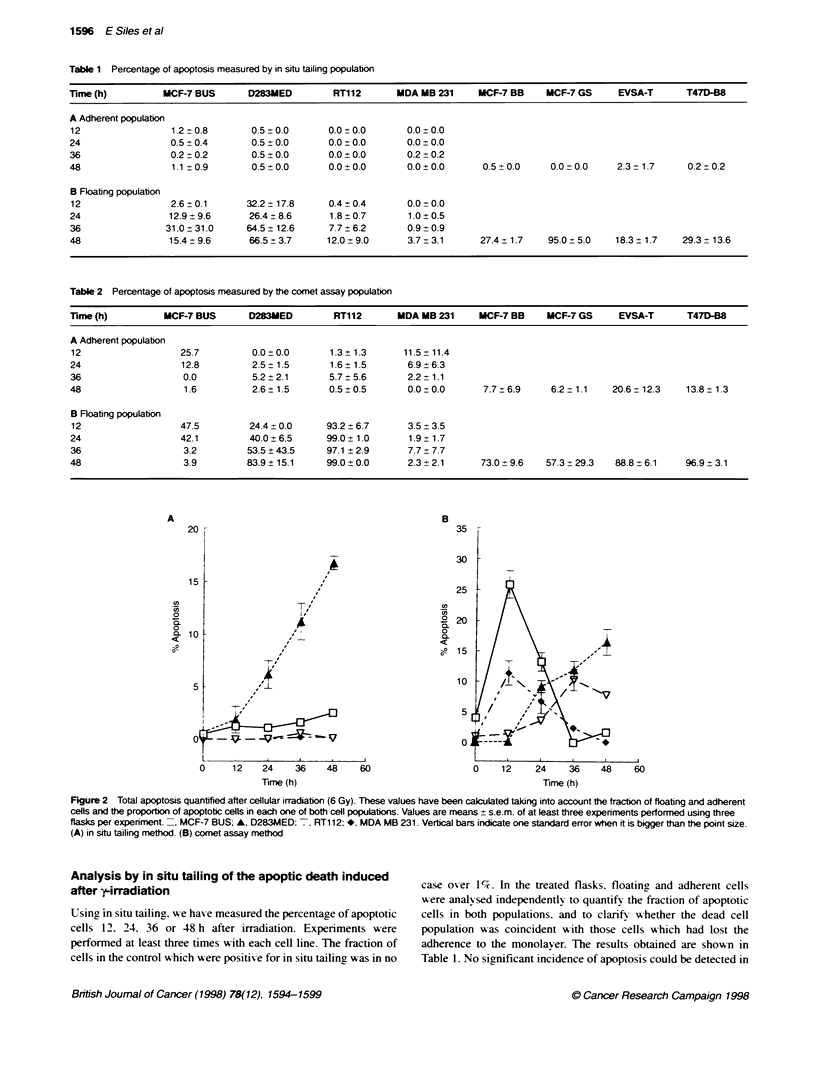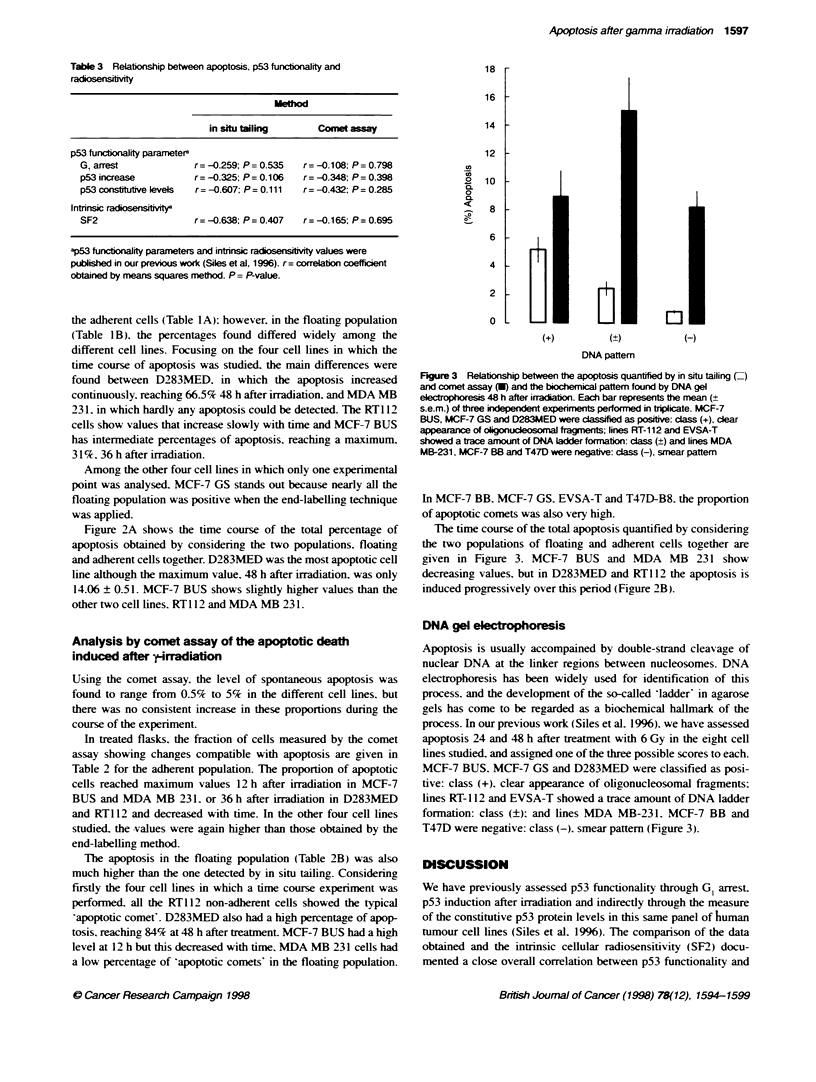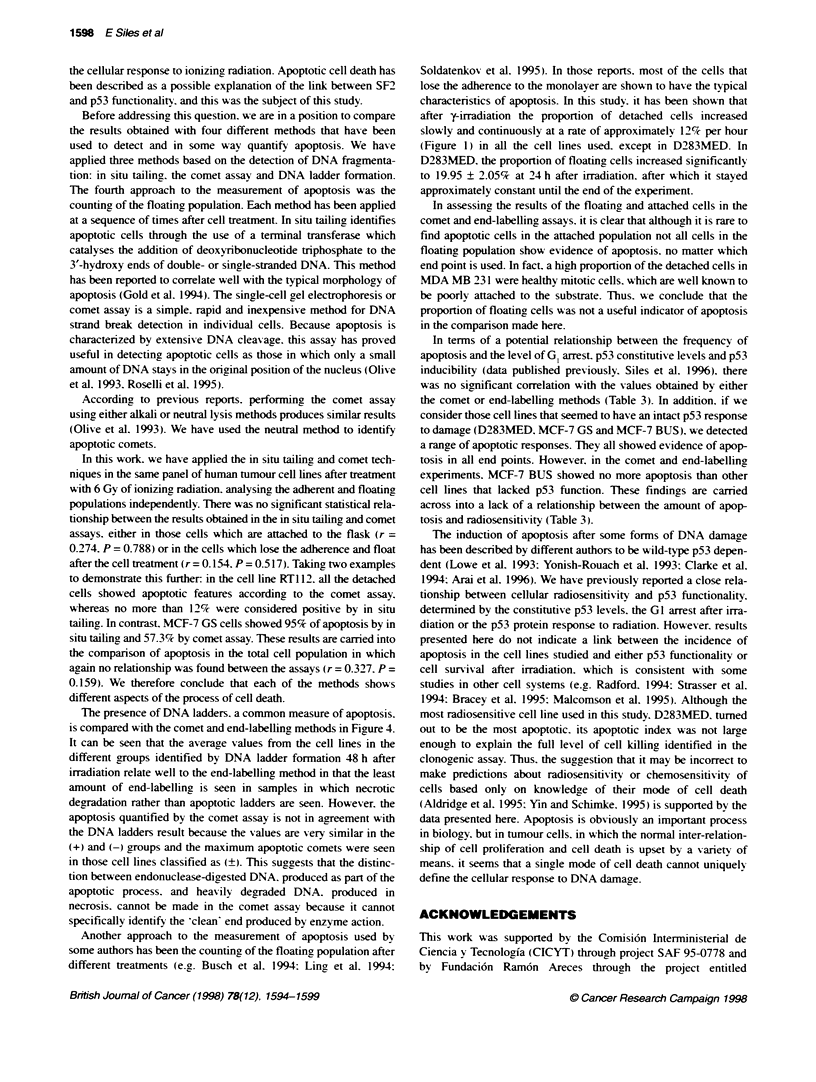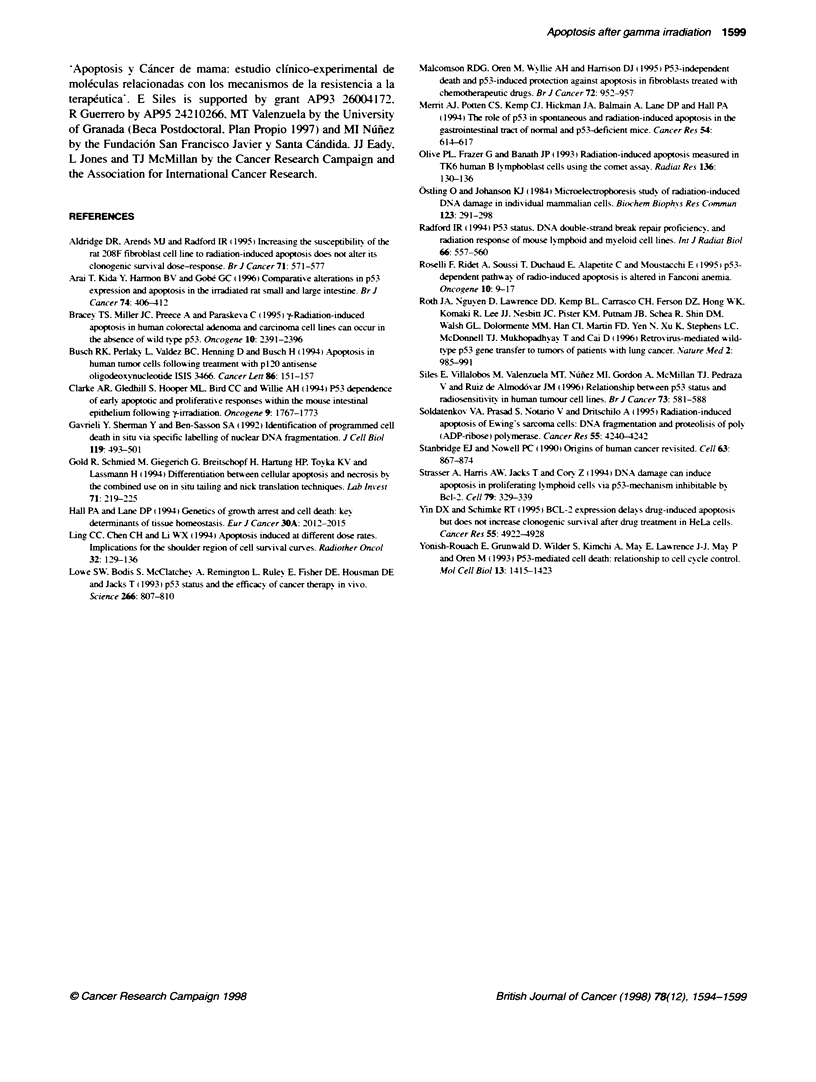# Apoptosis after gamma irradiation. Is it an important cell death modality?

**DOI:** 10.1038/bjc.1998.728

**Published:** 1998-12

**Authors:** E. Siles, M. Villalobos, L. Jones, R. Guerrero, J. J. Eady, M. T. Valenzuela, M. I. Núñez, T. J. McMillan, J. M. Ruiz de Almodóvar

**Affiliations:** Departamento de Radiología y Medicina Física, Facultad de Medicina, Universidad de Granada, Spain.

## Abstract

Apoptosis and necrosis are two different forms of cell death that can be induced by cytotoxic stress, such as ionizing radiation. We have studied the importance of apoptotic death induced after treatment with 6 Gy of gamma-irradiation in a panel of eight human tumour cell lines of different radiosensitivities. Three different techniques based on the detection of DNA fragmentation have been used, a qualitative one--DNA ladder formation --and two quantitative approaches--in situ tailing and comet assay. No statistically significant relationship between the two quantitative assays was found (r= 0.327, P = 0.159) so these methods seem to show different aspects of the process of cell death. The presence of the DNA ladder related well to the end-labelling method in that the least amount of end labelling was seen in samples in which necrotic degradation rather than apoptotic ladders were seen. However, as the results obtained by the comet assay are not in agreement with the DNA ladder experiments, we suggest that the distinction between the degraded DNA produced by apoptosis and necrosis may be difficult by this technique. Finally, although apoptosis has been proposed to be dependent on p53 functionality, and this may explain differences in cellular radiosensitivity, no statistically significant relationship was found between these parameters and apoptosis in the eight cell lines studied.


British Journal of Cancer (1998) 78(12), 1594–1599
© 1998 Cancer Research Campaign

### Apoptosis after gamma irradiation. Is it an important cell death modality?

**E Siles[1], M Villalobos[1], L Jones[2], R Guerrero[1], JJ Eady[2], MT Valenzuela[1], MI Núñez[1], TJ McMillan[3] and JM Ruiz de Almodóvar[1]**

[1]Laboratorio de Investigaciones Médicas y Biología Tumoral, Departamento de Radiología y Medicina Física, Facultad de Medicina, Universidad de Granada, 18071 Granada, Spain; [2]Radiotherapy Research Unit, Institute of Cancer Research, 15 Cotswold Road, Sutton, Surrey SM2 5NG, UK; [3]Department of Biological Sciences, Institute of Environmental and Natural Sciences, Lancaster University, Lancaster LA1 4YQ, UK

**Summary** Apoptosis and necrosis are two different forms of cell death that can be induced by cytotoxic stress, such as ionizing radiation. We have studied the importance of apoptotic death induced after treatment with 6 Gy of γ-irradiation in a panel of eight human tumour cell lines of different radiosensitivities. Three different techniques based on the detection of DNA fragmentation have been used, a qualitative one – DNA ladder formation – and two quantitative approaches – in situ tailing and comet assay. No statistically significant relationship between the two quantitative assays was found ($r = 0.327$, $P = 0.159$) so these methods seem to show different aspects of the process of cell death. The presence of the DNA ladder related well to the end-labelling method in that the least amount of end labelling was seen in samples in which necrotic degradation rather than apoptotic ladders were seen. However, as the results obtained by the comet assay are not in agreement with the DNA ladder experiments, we suggest that the distinction between the degraded DNA produced by apoptosis and necrosis may be difficult by this technique. Finally, although apoptosis has been proposed to be dependent on p53 functionality, and this may explain differences in cellular radiosensitivity, no statistically significant relationship was found between these parameters and apoptosis in the eight cell lines studied.

**Keywords:** apoptosis; radiosensitivity; cell cycle checkpoints; comet assay; in situ tailing; p53 functionality

Cell homeostasis is regulated by a balance between proliferation, growth arrest and cell death. Until recently, studies on oncogenesis have focused on the regulation of cell proliferation (Stanbridge and Nowell, 1990), however it is now clear that our view of neoplasia should include the concepts of regulation of growth arrest and cell death (Hall and Lane, 1994).

Two different forms of cell death have been described, apoptosis and necrosis, which can be distinguished by the distinctive changes that take place within the affected cells. Necrosis is a pathological form of cell death usually caused by an acute cellular injury; it is typified by irregular clumping of chromatin without significant change in its distribution, rapid cell swelling and lysis. In contrast, apoptosis is characterized by the early activation of endogenous proteases leading to cytoskeletal disruption, cell shrinkage, membrane blebbing and by the degradation of the DNA into fragments the size of oligonucleosomes.

Although both modalities can be induced by cytotoxic stress, there are several cellular factors that determine the nature of growth arrest and the type of cellular death in response to ionizing radiation. Among them, the tumour-suppressor protein, p53, is known to be essential for apoptosis after γ-irradiation (Lowe et al, 1993; Merrit et al, 1994, Arai et al, 1996), and its normal wild-type protein product can act to block the progression or the survival of cells that have sustained genetic damage. These events promoted by the p53 pathway avoid the rise of pools of aberrant surviving cells and seem to be indicative of a good therapeutic response to radiation or chemotherapeutic drugs in some tumour types (Roth et al, 1996). For example, we have previously examined the relationship between p53 status and radiosensitivity in eight human tumour cell lines that differed widely in their clonogenic survival after radiation. Our conclusion was that the constitutive p53 levels, $G_1$ arrest after irradiation or the p53 protein response to radiation may be good predictive tests for radiosensitivity in some cells (Siles et al, 1996). The main aim of the present study was to analyse the importance of apoptotic death after cellular irradiation and its relationship with p53 functionality and radiosensitivity in these human tumour cell lines.

*Received 28 October 1997*
*Revised 27 January 1998*
*Accepted 8 May 1998*

*Correspondence to:* JM Ruiz de Almodóvar, Departamento de Radiología y Medicina Física, Facultad de Medicina, Universidad de Granada, 18071 Granada, Spain

### MATERIALS AND METHODS

### Cell culture and radiation treatment

Eight human tumour cell lines have been studied in this work. They all have been described previously (Siles et al, 1996). Six of them were derived from human breast cancer (MCF-7 clones BB, BUS and GS, T47D, EVSA-T, MDA MB-231), RT112 from a human bladder carcinoma and D283MED is a human medulloblastoma cell line. This set of cells has been divided into two groups depending on the functionality of p53 protein assessed after irradiation: MCF-7 BUS, MCF-7 GS and D283MED with functional p53 protein and RT112, MDA MB 231, MCF-7 BB, EVSA-T and T47D with non-functional p53 protein.

Cell lines were grown in 10% fetal bovine serum-supplemented Dulbecco's modified Eagle medium (FBS-DMEM) (PAA-Laboratories, Linz, Austria) with penicillin (100 U $ml^{-1}$) and streptomycin (0.1 mg $ml^{-1}$). Cells were incubated at 37°C in a humidified atmosphere of 5% carbon dioxide/95% air. Freedom from mycoplasma contamination was checked regularly by testing with Hoechst 33528 dye (Sigma, St. Louis, MO, USA).

Cells in the exponential growth phase were irradiated using a $^{60}$Co source at a dose rate of 1.67 Gy min$^{-1}$. For apoptosis time course experiments after cellular irradiation, a single 6-Gy dose was delivered.

### Assay for DNA fragmentation

At the end of each incubation period after radiation, both floating and adherent cells were collected together, centrifuged for 10 min at 900 *g* and washed with phosphate-buffered saline (PBS). The pellet was resuspended in 600 µl of lysis buffer (100 mM tris-HCl, pH 8.0, 10 mM EDTA, 10 mM sodium chloride, 2% SDS and 10 µl of a 10 mg ml$^{-1}$ solution of RNAase), and incubated at 37°C for 30 min. We then added 100 µg ml$^{-1}$ proteinase and the mixture was incubated at 37°C overnight. The DNA was extracted by phenol and chloroform/isoamyl alcohol (24:1), precipitated overnight in −20°C ethanol containing sodium acetate at a final concentration of 0.3 M and centrifuged for 10 min, 4°C, at 12 000 r.p.m. (Microfuge, Beckman). The pellet of DNA was resuspended in Tris–EDTA buffer (0.1 M tris-HCl, pH 8.0, 10 mM EDTA). The DNA samples (0.2 µg each) were electrophoretically separated in 1% agarose gel containing ethidium bromide (0.5 µg ml$^{-1}$). DNA was visualized with a UV transilluminator, and the gels were photographed.

### In situ tailing

To quantify the amount of apoptotic cell death, the in situ tailing technique in which terminal deoxynucleotidyl transferase (TdT) incorporates nucleotides onto fragmented DNA of apoptotic cells (Gabrieli et al, 1992; Gold et al, 1994) has been applied as follows: at various time points after irradiation, floating and adherent cells were collected, counted and analysed separately. Cells were resuspended in medium at a final concentration of $2 \times 10^4$–$2 \times 10^5$ cells ml$^{-1}$. Cytospin preparations were made by adding 0.5 ml of each sample to a slide chamber and spinning for 10 min at 500 r.p.m. Slides were then fixed for 15 min on ice with 1% buffered formaldehyde in PBS (pH 7.4), washed in PBS and transferred to ice-cold 70% ethanol for 1 h. To analyse the samples, slides were rehydrated in PBS, excess fluid removed and 15 µl of TdT mixture (0.4 µl of 25 U µl$^{-1}$ TdT, 10 µl of 25 mM cobalt chloride, 20 µl × 5 terminal transferase reaction buffer, 1 µl of 0.5 nM biotin 16-dUTP and 77.6 µl of PBS from the kit Terminal Transferase, Boehringer Mannheim) pipetted over the cytospin preparation and sealed with a small piece of plastic coverslip. Slides were then placed in a humidifying hybridization chamber at 37°C for 30 min. Afterwards, slides were rinsed in PBS, dried and 20 µl of fluorescein-labelled avidin (Oncor, Gaithersburg, MD, USA) was added before reincubation for a further 30 min in the dark. Finally, slides were rinsed in three washes of PBD (phosphate-buffered detergent, Oncor) for 2 min, stained with propidium iodide antifade (Oncor), covered with a cover slip and visualized with a fluorescence microscope. Results were expressed as the percentage of apoptotic cells, stained in green, found in the adherent, floating or total cell populations. Each experiment was performed at least in triplicate.

### Comet assay

The second assay used to quantify apoptosis was the single-cell gel electrophoresis or comet assay, which was developed for detecting DNA strand breaks in individual cells (Ostling and Johanson, 1984). The extensive DNA fragmentation induced during apoptosis results in almost total DNA migration from the position of the nucleus in the comet assay, unlike fragmentation induced directly by radiation which, at the doses used in this study, only results in minor DNA migration. The comet assay was performed in neutral conditions. At different times after cell irradiation, floating and adherent cells were collected independently and 90-µl aliquots of $5 \times 10^4$ cells ml$^{-1}$ in PBS were embedded in 210 µl of 1% low-melting-point agarose, spread on fully frosted slides previously treated with 150 µl of normal-melting-point agarose, and lysed for 1 h at 4°C and 12 h at 37°C in lysis buffer (30 mM disodium EDTA, 0.5% SDS and 0.25 mg ml$^{-1}$ proteinase K, pH 8.0). Slides were then rinsed by immersion in TBE buffer 0.5 × (pH = 8.3), before electrophoresis in that same buffer (4°C, 1 V cm$^{-1}$, 25 min). Finally, slides were stained with ethidium bromide (20 µg µl$^{-1}$). Apoptotic figures were detected visually by scoring undamaged and extensively damaged cells present on coded slides and quantified independently as the percentage in the floating and adherent populations respectively. The total apoptotic percentage was also estimated by considering both populations together. Each experiment was performed at least in triplicate.

**Figure 1** Kinetics of cell detachment after cellular irradiation with 6 Gy. The experimental points corresponding to MCF-7 BUS, RT122 and MDA MB 231 have been fitted by lineal regression to a straight line (slope = 0.126 ± 0.017; $r = 0.897$; $P < 0.0001$). Errors bars represent the standard deviations of three independent experiments

### RESULTS

### Loss of cellular adherence

The quantification of the cells floating in the medium at different times after irradiation with a single dose has been used as an indicator of cell death by apoptosis (Ling et al, 1994). Figure 1 shows the kinetics of the loss of adherence in the different cell lines studied. Most of the cells show a progressive increase in the floating population with time after treatment. The experimental points, corresponding to MCF-7 BUS, RT112 and MDA MB 231 cell lines, plotted draw a linear relationship between the detached cell number and the time after radiation exposure (slope = 0.126 ± 0.017; correlation coefficient, $r = 0.897$; and $P < 0.0001$).

However, D283MED shows a different pattern of loss of cellular adherence, with a more rapid and more marked loss of attachment.

Table 1 Percentage of apoptosis measured by in situ tailing population

| Time (h) | MCF-7 BUS | D283MED | RT112 | MDA MB 231 | MCF-7 BB | MCF-7 GS | EVSA-T | T47D-B8 |
|---|---|---|---|---|---|---|---|---|
| **A** Adherent population | | | | | | | | |
| 12 | 1.2 ± 0.8 | 0.5 ± 0.0 | 0.0 ± 0.0 | 0.0 ± 0.0 | | | | |
| 24 | 0.5 ± 0.4 | 0.5 ± 0.0 | 0.0 ± 0.0 | 0.0 ± 0.0 | | | | |
| 36 | 0.2 ± 0.2 | 0.5 ± 0.0 | 0.0 ± 0.0 | 0.2 ± 0.2 | | | | |
| 48 | 1.1 ± 0.9 | 0.5 ± 0.0 | 0.0 ± 0.0 | 0.0 ± 0.0 | 0.5 ± 0.0 | 0.0 ± 0.0 | 2.3 ± 1.7 | 0.2 ± 0.2 |
| **B** Floating population | | | | | | | | |
| 12 | 2.6 ± 0.1 | 32.2 ± 17.8 | 0.4 ± 0.4 | 0.0 ± 0.0 | | | | |
| 24 | 12.9 ± 9.6 | 26.4 ± 8.6 | 1.8 ± 0.7 | 1.0 ± 0.5 | | | | |
| 36 | 31.0 ± 31.0 | 64.5 ± 12.6 | 7.7 ± 6.2 | 0.9 ± 0.9 | | | | |
| 48 | 15.4 ± 9.6 | 66.5 ± 3.7 | 12.0 ± 9.0 | 3.7 ± 3.1 | 27.4 ± 1.7 | 95.0 ± 5.0 | 18.3 ± 1.7 | 29.3 ± 13.6 |

Table 2 Percentage of apoptosis measured by the comet assay population

| Time (h) | MCF-7 BUS | D283MED | RT112 | MDA MB 231 | MCF-7 BB | MCF-7 GS | EVSA-T | T47D-B8 |
|---|---|---|---|---|---|---|---|---|
| **A** Adherent population | | | | | | | | |
| 12 | 25.7 | 0.0 ± 0.0 | 1.3 ± 1.3 | 11.5 ± 11.4 | | | | |
| 24 | 12.8 | 2.5 ± 1.5 | 1.6 ± 1.5 | 6.9 ± 6.3 | | | | |
| 36 | 0.0 | 5.2 ± 2.1 | 5.7 ± 5.6 | 2.2 ± 1.1 | | | | |
| 48 | 1.6 | 2.6 ± 1.5 | 0.5 ± 0.5 | 0.0 ± 0.0 | 7.7 ± 6.9 | 6.2 ± 1.1 | 20.6 ± 12.3 | 13.8 ± 1.3 |
| **B** Floating population | | | | | | | | |
| 12 | 47.5 | 24.4 ± 0.0 | 93.2 ± 6.7 | 3.5 ± 3.5 | | | | |
| 24 | 42.1 | 40.0 ± 6.5 | 99.0 ± 1.0 | 1.9 ± 1.7 | | | | |
| 36 | 3.2 | 53.5 ± 43.5 | 97.1 ± 2.9 | 7.7 ± 7.7 | | | | |
| 48 | 3.9 | 83.9 ± 15.1 | 99.0 ± 0.0 | 2.3 ± 2.1 | 73.0 ± 9.6 | 57.3 ± 29.3 | 88.8 ± 6.1 | 96.9 ± 3.1 |

**Figure 2** Total apoptosis quantified after cellular irradiation (6 Gy). These values have been calculated taking into account the fraction of floating and adherent cells and the proportion of apoptotic cells in each one of both cell populations. Values are means ± s.e.m. of at least three experiments performed using three flasks per experiment. □, MCF-7 BUS; ▲, D283MED; ▽, RT112; ◆, MDA MB 231. Vertical bars indicate one standard error when it is bigger than the point size. (**A**) in situ tailing method. (**B**) comet assay method

### Analysis by in situ tailing of the apoptic death induced after γ-irradiation

Using in situ tailing, we have measured the percentage of apoptotic cells 12, 24, 36 or 48 h after irradiation. Experiments were performed at least three times with each cell line. The fraction of cells in the control which were positive for in situ tailing was in no case over 1%. In the treated flasks, floating and adherent cells were analysed independently to quantify the fraction of apoptotic cells in both populations, and to clarify whether the dead cell population was coincident with those cells which had lost the adherence to the monolayer. The results obtained are shown in Table 1. No significant incidence of apoptosis could be detected in

 © Cancer Research Campaign 1998

**Table 3** Relationship between apoptosis, p53 functionality and radiosensitivity

| | Method | |
|---|---|---|
| | in situ tailing | Comet assay |
| p53 functionality parameter[a] | | |
| $G_1$ arrest | $r = -0.259$; $P = 0.535$ | $r = -0.108$; $P = 0.798$ |
| p53 increase | $r = -0.325$; $P = 0.106$ | $r = -0.348$; $P = 0.398$ |
| p53 constitutive levels | $r = -0.607$; $P = 0.111$ | $r = -0.432$; $P = 0.285$ |
| Intrinsic radiosensitivity[a] | | |
| SF2 | $r = -0.638$; $P = 0.407$ | $r = -0.165$; $P = 0.695$ |

[a]p53 functionality parameters and intrinsic radiosensitivity values were published in our previous work (Siles et al, 1996). *r* = correlation coefficient obtained by means squares method. *P* = *P*-value.

the adherent cells (Table 1A): however, in the floating population (Table 1B), the percentages found differed widely among the different cell lines. Focusing on the four cell lines in which the time course of apoptosis was studied, the main differences were found between D283MED, in which the apoptosis increased continuously, reaching 66.5% 48 h after irradiation, and MDA MB 231, in which hardly any apoptosis could be detected. The RT112 cells show values that increase slowly with time and MCF-7 BUS has intermediate percentages of apoptosis, reaching a maximum, 31%, 36 h after irradiation.

Among the other four cell lines in which only one experimental point was analysed, MCF-7 GS stands out because nearly all the floating population was positive when the end-labelling technique was applied.

Figure 2A shows the time course of the total percentage of apoptosis obtained by considering the two populations, floating and adherent cells together. D283MED was the most apoptotic cell line although the maximum value, 48 h after irradiation, was only 14.06 ± 0.51. MCF-7 BUS shows slightly higher values than the other two cell lines, RT112 and MDA MB 231.

### Analysis by comet assay of the apoptotic death induced after γ-irradiation

Using the comet assay, the level of spontaneous apoptosis was found to range from 0.5% to 5% in the different cell lines, but there was no consistent increase in these proportions during the course of the experiment.

In treated flasks, the fraction of cells measured by the comet assay showing changes compatible with apoptosis are given in Table 2 for the adherent population. The proportion of apoptotic cells reached maximum values 12 h after irradiation in MCF-7 BUS and MDA MB 231, or 36 h after irradiation in D283MED and RT112 and decreased with time. In the other four cell lines studied, the values were again higher than those obtained by the end-labelling method.

The apoptosis in the floating population (Table 2B) was also much higher than the one detected by in situ tailing. Considering firstly the four cell lines in which a time course experiment was performed, all the RT112 non-adherent cells showed the typical 'apoptotic comet'. D283MED also had a high percentage of apoptosis, reaching 84% at 48 h after treatment. MCF-7 BUS had a high level at 12 h but this decreased with time, MDA MB 231 cells had a low percentage of 'apoptotic comets' in the floating population.

**Figure 3** Relationship between the apoptosis quantified by in situ tailing (□) and comet assay (■) and the biochemical pattern found by DNA gel electrophoresis 48 h after irradiation. Each bar represents the mean (± s.e.m.) of three independent experiments performed in triplicate. MCF-7 BUS, MCF-7 GS and D283MED were classified as positive: class (+), clear appearance of oligonucleosomal fragments; lines RT-112 and EVSA-T showed a trace amount of DNA ladder formation: class (±) and lines MDA MB-231, MCF-7 BB and T47D were negative: class (–), smear pattern

In MCF-7 BB, MCF-7 GS, EVSA-T and T47D-B8, the proportion of apoptotic comets was also very high.

The time course of the total apoptosis quantified by considering the two populations of floating and adherent cells together are given in Figure 3. MCF-7 BUS and MDA MB 231 show decreasing values, but in D283MED and RT112 the apoptosis is induced progressively over this period (Figure 2B).

### DNA gel electrophoresis

Apoptosis is usually accompained by double-strand cleavage of nuclear DNA at the linker regions between nucleosomes. DNA electrophoresis has been widely used for identification of this process, and the development of the so-called 'ladder' in agarose gels has come to be regarded as a biochemical hallmark of the process. In our previous work (Siles et al, 1996), we have assessed apoptosis 24 and 48 h after treatment with 6 Gy in the eight cell lines studied, and assigned one of the three possible scores to each. MCF-7 BUS, MCF-7 GS and D283MED were classified as positive: class (+), clear appearance of oligonucleosomal fragments: lines RT-112 and EVSA-T showed a trace amount of DNA ladder formation: class (±): and lines MDA MB-231, MCF-7 BB and T47D were negative: class (–), smear pattern (Figure 3).

### DISCUSSION

We have previously assessed p53 functionality through $G_1$ arrest, p53 induction after irradiation and indirectly through the measure of the constitutive p53 protein levels in this same panel of human tumour cell lines (Siles et al, 1996). The comparison of the data obtained and the intrinsic cellular radiosensitivity (SF2) documented a close overall correlation between p53 functionality and

© Cancer Research Campaign 1998 

the cellular response to ionizing radiation. Apoptotic cell death has been described as a possible explanation of the link between SF2 and p53 functionality, and this was the subject of this study.

Before addressing this question, we are in a position to compare the results obtained with four different methods that have been used to detect and in some way quantify apoptosis. We have applied three methods based on the detection of DNA fragmentation: in situ tailing, the comet assay and DNA ladder formation. The fourth approach to the measurement of apoptosis was the counting of the floating population. Each method has been applied at a sequence of times after cell treatment. In situ tailing identifies apoptotic cells through the use of a terminal transferase which catalyses the addition of deoxyribonucleotide triphosphate to the 3′-hydroxy ends of double- or single-stranded DNA. This method has been reported to correlate well with the typical morphology of apoptosis (Gold et al. 1994). The single-cell gel electrophoresis or comet assay is a simple, rapid and inexpensive method for DNA strand break detection in individual cells. Because apoptosis is characterized by extensive DNA cleavage, this assay has proved useful in detecting apoptotic cells as those in which only a small amount of DNA stays in the original position of the nucleus (Olive et al. 1993, Roselli et al. 1995).

According to previous reports, performing the comet assay using either alkali or neutral lysis methods produces similar results (Olive et al. 1993). We have used the neutral method to identify apoptotic comets.

In this work, we have applied the in situ tailing and comet techniques in the same panel of human tumour cell lines after treatment with 6 Gy of ionizing radiation, analysing the adherent and floating populations independently. There was no significant statistical relationship between the results obtained in the in situ tailing and comet assays, either in those cells which are attached to the flask ($r$ = 0.274, $P$ = 0.788) or in the cells which lose the adherence and float after the cell treatment ($r$ = 0.154, $P$ = 0.517). Taking two examples to demonstrate this further: in the cell line RT112, all the detached cells showed apoptotic features according to the comet assay, whereas no more than 12% were considered positive by in situ tailing. In contrast, MCF-7 GS cells showed 95% of apoptosis by in situ tailing and 57.3% by comet assay. These results are carried into the comparison of apoptosis in the total cell population in which again no relationship was found between the assays ($r$ = 0.327, $P$ = 0.159). We therefore conclude that each of the methods shows different aspects of the process of cell death.

The presence of DNA ladders, a common measure of apoptosis, is compared with the comet and end-labelling methods in Figure 4. It can be seen that the average values from the cell lines in the different groups identified by DNA ladder formation 48 h after irradiation relate well to the end-labelling method in that the least amount of end-labelling is seen in samples in which necrotic degradation rather than apoptotic ladders are seen. However, the apoptosis quantified by the comet assay is not in agreement with the DNA ladders result because the values are very similar in the (+) and (−) groups and the maximum apoptotic comets were seen in those cell lines classified as (±). This suggests that the distinction between endonuclease-digested DNA, produced as part of the apoptotic process, and heavily degraded DNA, produced in necrosis, cannot be made in the comet assay because it cannot specifically identify the 'clean' end produced by enzyme action.

Another approach to the measurement of apoptosis used by some authors has been the counting of the floating population after different treatments (e.g. Busch et al. 1994; Ling et al. 1994; Soldatenkov et al. 1995). In those reports, most of the cells that lose the adherence to the monolayer are shown to have the typical characteristics of apoptosis. In this study, it has been shown that after γ-irradiation the proportion of detached cells increased slowly and continuously at a rate of approximately 12% per hour (Figure 1) in all the cell lines used, except in D283MED. In D283MED, the proportion of floating cells increased significantly to 19.95 ± 2.05% at 24 h after irradiation, after which it stayed approximately constant until the end of the experiment.

In assessing the results of the floating and attached cells in the comet and end-labelling assays, it is clear that although it is rare to find apoptotic cells in the attached population not all cells in the floating population show evidence of apoptosis, no matter which end point is used. In fact, a high proportion of the detached cells in MDA MB 231 were healthy mitotic cells, which are well known to be poorly attached to the substrate. Thus, we conclude that the proportion of floating cells was not a useful indicator of apoptosis in the comparison made here.

In terms of a potential relationship between the frequency of apoptosis and the level of $G_1$ arrest, p53 constitutive levels and p53 inducibility (data published previously, Siles et al. 1996), there was no significant correlation with the values obtained by either the comet or end-labelling methods (Table 3). In addition, if we consider those cell lines that seemed to have an intact p53 response to damage (D283MED, MCF-7 GS and MCF-7 BUS), we detected a range of apoptotic responses. They all showed evidence of apoptosis in all end points. However, in the comet and end-labelling experiments, MCF-7 BUS showed no more apoptosis than other cell lines that lacked p53 function. These findings are carried across into a lack of a relationship between the amount of apoptosis and radiosensitivity (Table 3).

The induction of apoptosis after some forms of DNA damage has been described by different authors to be wild-type p53 dependent (Lowe et al. 1993; Yonish-Rouach et al. 1993; Clarke et al. 1994; Arai et al. 1996). We have previously reported a close relationship between cellular radiosensitivity and p53 functionality, determined by the constitutive p53 levels, the G1 arrest after irradiation or the p53 protein response to radiation. However, results presented here do not indicate a link between the incidence of apoptosis in the cell lines studied and either p53 functionality or cell survival after irradiation, which is consistent with some studies in other cell systems (e.g. Radford, 1994; Strasser et al. 1994; Bracey et al. 1995; Malcomson et al. 1995). Although the most radiosensitive cell line used in this study, D283MED, turned out to be the most apoptotic, its apoptotic index was not large enough to explain the full level of cell killing identified in the clonogenic assay. Thus, the suggestion that it may be incorrect to make predictions about radiosensitivity or chemosensitivity of cells based only on knowledge of their mode of cell death (Aldridge et al. 1995; Yin and Schimke, 1995) is supported by the data presented here. Apoptosis is obviously an important process in biology, but in tumour cells, in which the normal inter-relationship of cell proliferation and cell death is upset by a variety of means, it seems that a single mode of cell death cannot uniquely define the cellular response to DNA damage.

### ACKNOWLEDGEMENTS

This work was supported by the Comisión Interministerial de Ciencia y Tecnología (CICYT) through project SAF 95-0778 and by Fundación Ramón Areces through the project entitled

© Cancer Research Campaign 1998

'Apoptosis y Cáncer de mama: estudio clínico-experimental de moléculas relacionadas con los mecanismos de la resistencia a la terapéutica'. E Siles is supported by grant AP93 26004172. R Guerrero by AP95 24210266. MT Valenzuela by the University of Granada (Beca Postdoctoral. Plan Propio 1997) and MI Núñez by the Fundación San Francisco Javier y Santa Cándida. JJ Eady. L Jones and TJ McMillan by the Cancer Research Campaign and the Association for International Cancer Research.

### REFERENCES

Aldridge DR. Arends MJ and Radford IR (1995) Increasing the susceptibility of the rat 208F fibroblast cell line to radiation-induced apoptosis does not alter its clonogenic survival dose–response. *Br J Cancer* **71**: 571–577

Arai T. Kida Y. Harmon BV and Gobé GC (1996) Comparative alterations in p53 expression and apoptosis in the irradiated rat small and large intestine. *Br J Cancer* **74**: 406–412

Bracey TS. Miller JC. Preece A and Paraskeva C (1995) γ-Radiation-induced apoptosis in human colorectal adenoma and carcinoma cell lines can occur in the absence of wild type p53. *Oncogene* **10**: 2391–2396

Busch RK. Perlaky L. Valdez BC. Henning D and Busch H (1994) Apoptosis in human tumor cells following treatment with p120 antisense oligodeoxynucleotide ISIS 3466. *Cancer Lett* **86**: 151–157

Clarke AR. Gledhill S. Hooper ML. Bird CC and Willie AH (1994) P53 dependence of early apoptotic and proliferative responses within the mouse intestinal epithelium following γ-irradiation. *Oncogene* **9**: 1767–1773

Gavrieli Y. Sherman Y and Ben-Sasson SA (1992) Identification of programmed cell death in situ via specific labelling of nuclear DNA fragmentation. *J Cell Biol* **119**: 493–501

Gold R. Schmied M. Giegerich G. Breitschopf H. Hartung HP. Toyka KV and Lassmann H (1994) Differentiation between cellular apoptosis and necrosis by the combined use on in situ tailing and nick translation techniques. *Lab Invest* **71**: 219–225

Hall PA and Lane DP (1994) Genetics of growth arrest and cell death: key determinants of tissue homeostasis. *Eur J Cancer* **30A**: 2012–2015

Ling CC. Chen CH and Li WX (1994) Apoptosis induced at different dose rates. Implications for the shoulder region of cell survival curves. *Radiother Oncol* **32**: 129–136

Lowe SW. Bodis S. McClatchey A. Remington L. Ruley E. Fisher DE. Housman DE and Jacks T (1993) p53 status and the efficacy of cancer therapy in vivo. *Science* **266**: 807–810

Malcomson RDG. Oren M. Wyllie AH and Harrison DJ (1995) P53-independent death and p53-induced protection against apoptosis in fibroblasts treated with chemotherapeutic drugs. *Br J Cancer* **72**: 952–957

Merrit AJ. Potten CS. Kemp CJ. Hickman JA. Balmain A. Lane DP and Hall PA (1994) The role of p53 in spontaneous and radiation-induced apoptosis in the gastrointestinal tract of normal and p53-deficient mice. *Cancer Res* **54**: 614–617

Olive PL. Frazer G and Banath JP (1993) Radiation-induced apoptosis measured in TK6 human B lymphoblast cells using the comet assay. *Radiat Res* **136**: 130–136

Östling O and Johanson KJ (1984) Microelectrophoresis study of radiation-induced DNA damage in individual mammalian cells. *Biochem Biophys Res Commun* **123**: 291–298

Radford IR (1994) P53 status. DNA double-strand break repair proficiency. and radiation response of mouse lymphoid and myeloid cell lines. *Int J Radiat Biol* **66**: 557–560

Roselli F. Ridet A. Soussi T. Duchaud E. Alapetite C and Moustacchi E (1995) p53-dependent pathway of radio-induced apoptosis is altered in Fanconi anemia. *Oncogene* **10**: 9–17

Roth JA. Nguyen D. Lawrence DD. Kemp BL. Carrasco CH. Ferson DZ. Hong WK. Komaki R. Lee JJ. Nesbitt JC. Pister KM. Putnam JB. Schea R. Shin DM. Walsh GL. Dolormente MM. Han CI. Martin FD. Yen N. Xu K. Stephens LC. McDonnell TJ. Mukhopadhyay T and Cai D (1996) Retrovirus-mediated wild-type p53 gene transfer to tumors of patients with lung cancer. *Nature Med* **2**: 985–991

Siles E. Villalobos M. Valenzuela MT. Núñez MI. Gordon A. McMillan TJ. Pedraza V and Ruiz de Almodóvar JM (1996) Relationship between p53 status and radiosensitivity in human tumour cell lines. *Br J Cancer* **73**: 581–588

Soldatenkov VA. Prasad S. Notario V and Dritschilo A (1995) Radiation-induced apoptosis of Ewing's sarcoma cells: DNA fragmentation and proteolisis of poly (ADP-ribose) polymerase. *Cancer Res* **55**: 4240–4242

Stanbridge EJ and Nowell PC (1990) Origins of human cancer revisited. *Cell* **63**: 867–874

Strasser A. Harris AW. Jacks T and Cory Z (1994) DNA damage can induce apoptosis in proliferating lymphoid cells via p53-mechanism inhibitable by Bcl-2. *Cell* **79**: 329–339

Yin DX and Schimke RT (1995) BCL-2 expression delays drug-induced apoptosis but does not increase clonogenic survival after drug treatment in HeLa cells. *Cancer Res* **55**: 4922–4928

Yonish-Rouach E. Grunwald D. Wilder S. Kimchi A. May E. Lawrence J-J. May P and Oren M (1993) P53-mediated cell death: relationship to cell cycle control. *Mol Cell Biol* **13**: 1415–1423

© Cancer Research Campaign 1998